# Fluid type influences acute hydration and muscle performance recovery in human subjects

**DOI:** 10.1186/s12970-019-0282-y

**Published:** 2019-04-04

**Authors:** Preston R. Harris, Douglas A. Keen, Eleni Constantopoulos, Savanna N. Weninger, Eric Hines, Matthew P. Koppinger, Zain I. Khalpey, John P. Konhilas

**Affiliations:** 1 Department of Nutritional Sciences, University of Arizona, Tucson, AZ 85721 USA; 2 Department of Physiology, University of Arizona, Tucson, AZ 85721 USA; 3 Sarver Molecular Cardiovascular Research Program, University of Arizona, Tucson, AZ 85724 USA; 4 Department of Surgery, University of Arizona, Tucson, AZ 85721 USA

**Keywords:** Dehydration, Exercise, Deep sea water, Humans, Sweat

## Abstract

**Background:**

Exercise and heat trigger dehydration and an increase in extracellular fluid osmolality, leading to deficits in exercise performance and thermoregulation. Evidence from previous studies supports the potential for deep-ocean mineral water to improve recovery of exercise performance post-exercise. We therefore wished to determine whether acute rehydration and muscle strength recovery was enhanced by deep-ocean mineral water following a dehydrating exercise, compared to a sports drink or mountain spring water. We hypothesized that muscle strength would decrease as a result of dehydrating exercise, and that recovery of muscle strength and hydration would depend on the type of rehydrating fluid.

**Methods:**

Using a counterbalanced, crossover study design, female (*n* = 8) and male (*n* = 9) participants performed a dehydrating exercise protocol under heat stress until achieving 3% body mass loss. Participants rehydrated with either deep-ocean mineral water (**Deep**), mountain spring water (**Spring**), or a carbohydrate-based sports drink (**Sports**) at a volume equal to the volume of fluid loss. We measured relative hydration using salivary osmolality (S_osm_) and muscle strength using peak torque from a leg extension maneuver.

**Results:**

S_osm_ significantly increased (*p* < 0.0001) with loss of body mass during the dehydrating exercise protocol. Males took less time (90.0 ± 18.3 min; *P* < 0.0034) to reach 3% body mass loss when compared to females (127.1 ± 20.0 min). We used a mono-exponential model to fit the return of S_osm_ to baseline values during the rehydrating phase. Whether fitting stimulated or unstimulated S_osm_, male and female participants receiving **Deep** as the hydrating fluid exhibited the most rapid return to baseline S_osm_ (p < 0.0001) regardless of the fit parameter. Males compared to females generated more peak torque (*p* = 0.0005) at baseline (308.3 ± 56.7 Nm vs 172.8 ± 40.8 Nm, respectively) and immediately following 3% body mass loss (276.3 ± 39.5 Nm vs 153.5 ± 35.9 Nm). Participants experienced a loss. We also identified a significant effect of rehydrating fluid and sex on post-rehydration peak torque (*p* < 0.0117).

**Conclusion:**

We conclude that deep-ocean mineral water positively affected hydration recovery after dehydrating exercise, and that it may also be beneficial for muscle strength recovery, although this, as well as the influence of sex, needs to be further examined by future research.

**Trial registration:**

clincialtrials.gov PRS, NCT02486224. Registered 08 June 2015.

## Background

The volume of total body water in humans represents approximately 60–70% of total body weight in men and 50–60% of body weight in women, with some variability primarily due to differences in body composition [1]. Many factors impact total fluid volume including diet (water and sodium intake), environmental temperature, evaporation, activity level, and certain disease states. Humans have a remarkable capacity to maintain constant osmolality of extracellular fluid (ECF) through both behavioral responses and physiological mechanisms that restore ECF to homeostatic values [2]. Still, loss of body water, or dehydration, impairs normal physiologic function. Dehydration increases cardiovascular strain by reducing blood volume through fluid loss, thereby decreasing stroke volume, and increasing heart rate. When core temperature increases as a result of exercise and dehydration, elevated skin blood flow displaces blood away from the central blood volume, exacerbating cardiovascular strain [3]. In addition to this increase in cutaneous blood flow, sweat production increases in an effort to dissipate heat to the environment through evaporative cooling [4].

Sweat glands produce a hypotonic fluid in relation to plasma by drawing fluid from the ECF concomitant with reabsorption of sodium and chlorine via the cystic fibrosis transmembrane protein (CFTR) channels [5]. This results in both a decrease in total body water volume and an increase in ECF osmolality [6]. Governed by hydrostatic, osmotic and oncotic pressures, the increase in ECF osmolality triggers water movement from plasma and intracellular stores to restore osmolality in the interstitial fluid compartment [1, 2]. Osmotic fluctuations, if severe enough, can have serious health consequences, such as weakness, cardiac arrest, spasticity, coma, seizures, and death [2, 7]. Furthermore, dehydration impairs thermoregulation [8] and exercise performance, independently of thermal, dietary, or metabolic stressors [3, 9]. Exercising in a hot environment amplifies dehydration, exacerbates the above effects, and accelerates performance deficits [3, 10, 11].

An obvious key to preventing the detrimental impact of fluid loss on exercise performance is to replenish fluid deficit through oral consumption [5]. Although the American College of Sports Medicine Guidelines on Nutrition and Athletic Performance recommend the amount of fluid intake, there is no clear endorsement regarding the specific type of rehydrating fluid [12]. A recent study shows that deep-ocean water taken from the coast of Hualien, Taiwan at a depth of 662 m improves recovery following a dehydrating exercise, evidenced by accelerated recovery of aerobic capacity, increased lower-body muscle power performance, and significantly reduced levels of exercise-induced muscle damage markers compared to participants drinking purified tap-water [13]. In another study, deep-ocean mineral water was shown to increase the exercise performance of gerbils, compared to distilled water, measured by retention rates during a 90-min treadmill exercise [14]. Considering the established connection between hydration status and exercise performance, these data suggest that deep-ocean mineral water may provide optimal rehydration for performance recovery following high-intensity exercise. Accordingly, data from our previous study [15] suggested that Kona Deep®-ocean mineral water had the potential to improve lower-body muscle strength as well as acute rehydration rate after dehydrating exercise.

Deep ocean mineral water has a mineral composition that differs significantly from that of water found on or near the surface, often containing much higher amounts of sodium, potassium, chloride, magnesium, and various other trace minerals which are not found in surface water sources. Although mineral profiles of deep ocean mineral water differ by specific site, it is this unique mineral composition that we believe is responsible for the effects described above, though we do not propose a mechanism here.

The objective of this study was to improve and expand upon the observations from our previous work, regarding the impact of fluid type on rehydration and muscle performance recovery following rehydration at the completion of a dehydrating exercise protocol under heat stress. We hypothesized that rehydration with Kona Deep® ocean mineral water will accelerate the rate of acute rehydration, and will improve muscle strength recovery compared to Gatorade® or mountain spring water. Secondarily, we make observations on potential sex differences in these parameters.

## Methods

### Participants

Participants eligible for this study included females and males ages 20–25 years, who were nonsmokers and were well-conditioned with moderate to excellent aerobic capacity. Participants were free from medication, stimulants, dietary supplements (including vitamins/minerals, amino acids, and herbal supplements, as defined by the FDA), or major health-related issues, as determined by a University of Arizona Health History Screening Questionnaire (UAHHSQ). Age-matched female and male study participants meeting eligibility criteria were further selected for equivalent fitness levels based on a self-reported 3–6 h of athletic conditioning per week. The study population engaged in primarily dynamic activities including cycling, running, hockey, soccer, and triathlons. With a conservative estimate of salivary osmolality at baseline (100 mmol/Kg) compared to peak (150 mmol/Kg) during the experimental protocol with a standard deviation of 30, sample size was determined using a power of 0.8 at α = 0.05 (6 or greater per group). This was also confirmed using power analysis of mean population serum and urinary osmolality. All participants provided consent under protocols adhering to guidelines approved by the Institutional Review Board at the University of Arizona and in accordance with the Declaration of Helsinki. Participants were asked to follow a normal diet while avoiding foods high in sodium 24 h prior to study initiation, and to avoid any exercise 24 h prior to each trial. Dietary data was not collected. In addition, participants were required to abstain from alcohol consumption 36 h prior to each exercise trial, as alcohol has diuretic effects, and may confound observed effects of dehydration. Female participants were asked to complete the study early in their menstrual cycle to avoid the potentially confounding issue of fluid retention. Participants were also instructed to begin each trial in a similarly hydrated state (confirmed by consistent baseline salivary osmolality at the start of each trial).

### Study design

We used a counterbalanced, crossover study design in which participants (*n* = 17) were randomized to begin in one of 3 experimental groups: Kona Deep® deep-ocean mineral water (**Deep**), Gatorade® sports drink (**Sports**), or Arrowhead mountain spring water (**Spring**) (See Table 1 for fluid nutrient comparison). Each participant was required to complete the dehydrating exercise protocol 3 times, one trial for each hydration fluid, each separated by a period of at least 48 h, to give participants adequate recovery time. After the first trial, participants were randomized to complete the second arm of the study, hydrating with one of the two remaining hydrating fluids. During the third trial, participants rehydrated with the last remaining fluid.

**Table 1:** Fluid comparison of selected nutrients

Nutrient	Kona Deep® (deep-ocean mineral water)^a^	Arrowhead (mountain spring water)^b^	Gatorade® (carbohydrate-based sports drink)^c^
Sodium	85 mg/L	3 mg/L	450.9 mg/L
Chloride	150 mg/L	0–12 mg/L	408.3 mg/L
Potassium	4 mg/L	0–2.9 mg/L	126.8 mg/L
Magnesium	4.3 mg/L	1.4 mg/L	0 mg/L
Calcium	1.4 mg/L	4 mg/L	0 mg/L
Boron	0.65 mg/L	0 mg/L	0 mg/L
Bromide	540 μg/L	3.1 μg/L	0 μg/L
Chromium	2.2 μg/L	0 μg/L	0 μg/L
Carbohydrates	0 g/L	0 g/L	59.2 g/L

^a^Values provided by Kona Deep® Corporation via a 3rd party laboratory report. ^b^Values are minimum ranges based on Arrowhead 2016 Water Analysis Report at www.nestle-watersna.com. ^c^Values calculated based on amounts reported for Gatorade® Thirst Quencher at www.Gatorade.com. Values were not validated in house

### Dehydration and hydration protocols

**Fig. 1:**
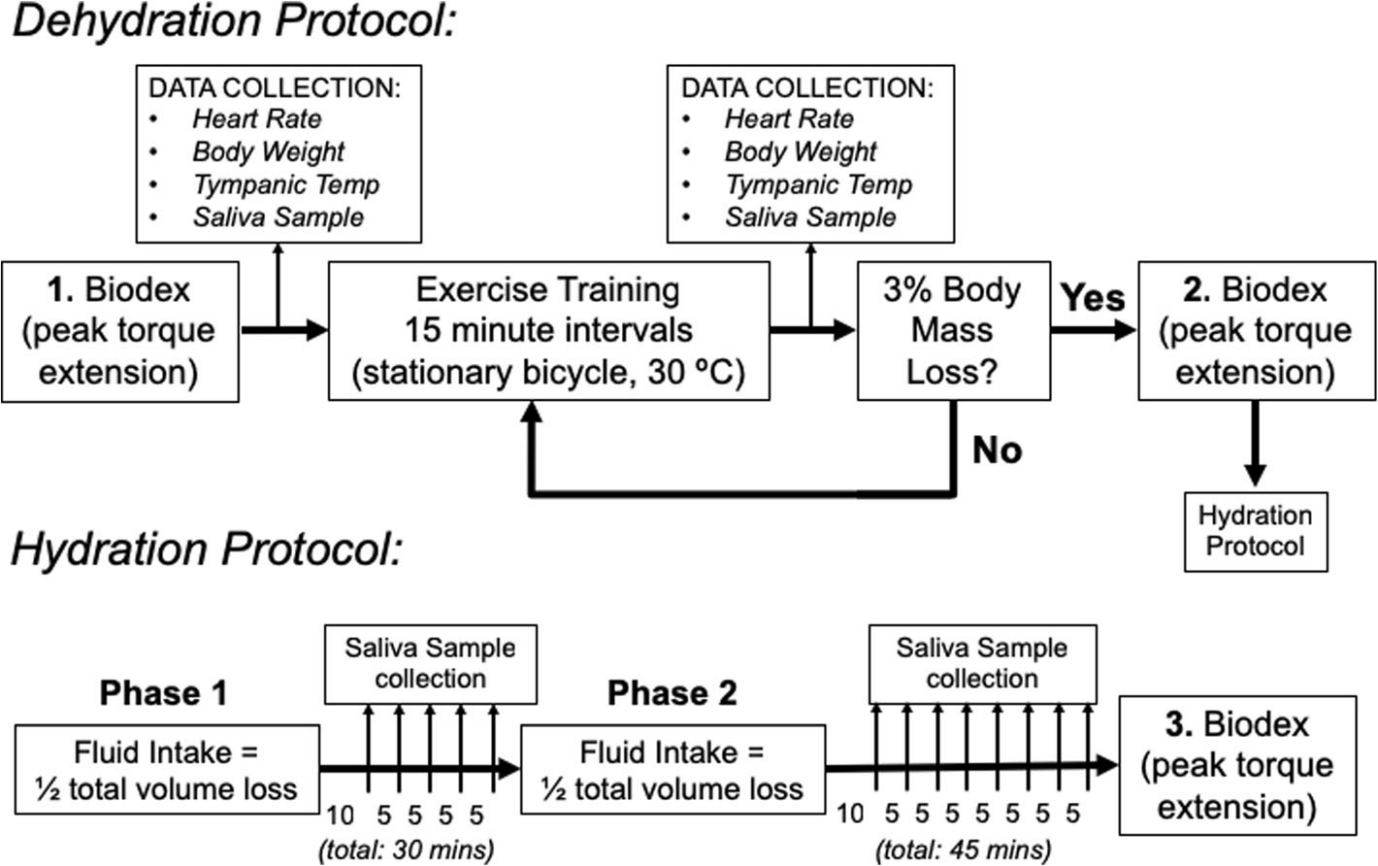
Experimental design and protocol. Dehydration Protocol: Euhydrated participants were randomly assigned in a counterbalanced fashion to one of three groups (Deep, Sports, or Spring). Prior to data collection, participants executed 1 of 3 peak torque extension maneuvers to obtain a **baseline** value. Following peak torque extension, parameters were collected (indicated in the panel labeled DATA COLLECTION) and exercise was initiated using a stationary bicycle under moderate heat stress (32–35 °C). Exercise was continued for 15 min followed by collection of parameters. If participants did not achieve 3% body mass loss, exercise was reinitiated for another 15 min. This cycle was continued until participants lost a minimum 3% of body mass and participants were not allowed to evacuate or intake any fluids. Upon completion of the Dehydration Protocol, participants immediately executed the second (2) of 3 peak torque extension maneuvers to obtain a **post-exercise** value and transitioned to the Hydration protocol. Hydration protocol: Participants rehydrated with 1 of 3 fluids, in 2 phases. **Phase 1**: Participants consumed fluids at ½ of the total volume lost. 10 min following fluid intake a saliva sample was collected. Sample collection continued at 5-min intervals until 30 min from the time of fluid intake. **Phase 2**: The remaining amount (½ of the total volume lost) of fluid was ingested followed by saliva sample collection 10 min later. Saliva collection continued at 5-min intervals until 45 min from the second fluid intake. Immediately following the final saliva collection, participants executed the third [3] of 3 peak torque extension maneuvers to obtain a **post-hydration** value.

A graphical summary of the Dehydration and Hydration Protocol is illustrated in Fig. 1. Participants were asked to remove any excess or loose clothing including shirts, athletic pants, shoes, and socks. Participants were instrumented with a Polar™ heart rate monitor allowing continuous monitoring of heart rate. Baseline measurements of heart rate, body weight (using a digital scale) and tympanic temperature (Braun ThermoScan® PRO 4000) were collected prior to the initiation of the exercise dehydration protocol. In addition, stimulated and unstimulated saliva samples (detailed below) were taken to establish baseline osmolality values and to ensure all participants were similarly hydrated at the start of each trial. Next, participants initiated exercise using a Monark® stationary cycle under moderate heat stress using heat lamps to provide warm conditions of about 30 °C, since sweat rate is greatest at warm ambient temperatures [16]. Participants were instructed to sustain about 60% of maximum heart rate and maintain 150–200 watts on the stationary bicycle at a cadence greater than 70 rpm. Participants self-monitored and adjusted watts by varying cycle speed or resistance. Exercise was continued for 15 min, at which time participants were asked to remove any excess or loose clothing as above, and towel-dry for data collection (body weight, heart rate, temperature, saliva samples). Participants were not allowed to evacuate or intake any fluids during the exercise protocol. This protocol was repeated at 15-min intervals until target dehydration, indicated by a body mass loss of 3% was achieved. Studies have indicated that greater than 2% body mass loss by dehydration results in a significant exercise deficit [3, 10, 13, 14].

When 3% body mass loss was reached, participants rehydrated by consuming a volume of fluid (**Deep**, **Sports**, or **Spring**) equal to their body mass loss, assuming that each 1 L = 1 kg. Rehydration occurred in two phases. In the first phase, participants consumed one-half of the total volume lost. Stimulated and unstimulated saliva samples were collected starting at 10 min after this initial rehydration phase (to avoid the cofounding effect on salivary osmolality due to oral rinse) [17], and at each subsequent 5-min interval, for 30 min. After this 30-min time period, the second phase occurred, in which participants consumed the remainder of the fluid. Saliva samples were taken starting at 10 min after this second rehydration phase, and at each subsequent 5-min interval, for 45 min.

### Lower body muscle performance

Peak torque of the left knee extensors, measured on a Biodex™ System 3 dynamometer, was used as a measure of lower body muscle performance. As illustrated in Fig. 1, a peak torque extension maneuver was executed three times during the Dehydration and Hydration Protocols: 1) **baseline** – prior to exercise, 2) **post-exercise** – following loss of 3% body mass, and 3) **post-hydration** – after the final saliva collection (75 min post-exercise). Participants performed a series of 3 maximal contractions of the left knee extensors at a rotational velocity of 60 degrees per second using the Biodex™. To maintain consistency, participants performed this test oriented in the same position, and using the same hand grips for support during each of the measurements. Participants were also vigorously encouraged to exert maximal effort on each measurement by the same individuals. To familiarize participants with the Biodex™ machine, they were allowed a “practice” period prior to each measurement to move their leg at will, and become accustomed to the speed of movement. When the participants were comfortable and ready to perform the measured test, they indicated this to the machine by holding their leg in a fully contracted position for several seconds, signaling the measurement process detailed above to proceed.

### Salivary osmolality

Hydration status was monitored using salivary osmolality. Several different measurements can be used to assess hydration, including serum, saliva, and urine osmolality, and urine volume and specific gravity, and the most appropriate measurement depends on the mode of dehydration, and the frequency of the measurement. Previous studies have demonstrated that, for repeated measurements during active dehydration (i.e. exercise) in the heat, salivary osmolality is an accurate, non-invasive method to measure ECF osmolality [18].

Saliva was collected from the oral cavity, first as a passive expectorant (unstimulated) [17, 19], and then following mechanical (stimulated) orofacial movement (chewing on a cotton swab). Stimulated saliva samples were spun down in a centrifuge for 10 min to collect saliva from the cotton swabs. All samples, both stimulated and unstimulated were then vortexed to homogenize the samples. Salivary osmolality was measured using a dedicated vapor pressure osmometer (Wescor VAPRO® 5600) by the same three individuals, using 10 μl of each sample, and each sample was run in triplicate. This was done immediately after sample collection to prevent sample spoilage. In addition to daily calibrations, the osmometer was calibrated prior to each new biological sample.

### Data and statistical analysis

All values are presented as mean (SD). Body Mass Index (BMI) was calculated using the following equation:

$$ \mathrm{BMI}=\mathrm{body}\ \mathrm{weight}\ \left(\mathrm{in}\ \mathrm{kg}\right)/\mathrm{height}\ {\left(\mathrm{in}\ \mathrm{meters}\right)}^2 $$

Body Surface Area (BSA) was calculated based on the following equation [20]:

$$ \mathrm{BSA}\ \left({\mathrm{m}}^2\right)=\surd \left(\mathrm{Height}\ \left(\mathrm{in}\ \mathrm{cm}\right)\ \mathrm{x}\ \mathrm{body}\ \mathrm{weight}\ \left(\mathrm{in}\ \mathrm{kg}\right)\right)/3600 $$

To compare heart rate, body weight (BW), BMI, BSA, and tympanic temperature at baseline and peak, the measured values in each individual were averaged across the three arms of the study. The same method was used to calculate mean values for time to 3% body mass loss, sweat rate, baseline and post-exercise peak torque. Salivary osmolality (S_osm_) was plotted against percent body mass loss; body mass loss was calculated as the difference in body mass after completion of the dehydrating exercise, from body mass at trial initiation. This value was divided by body mass at trial initiation and expressed as a percentage. For each individual, S_osm_ against body mass loss (%) was fit by linear regression. Differences in the slopes of the regression lines between the groups were calculated using one-way analysis of variance (ANOVA) with Bonferroni post hoc correction for multiple comparisons. The return of S_osm_ to baseline during the Hydration Protocol was best fit by a mono-exponential (one-phase decay) model where,

$$ \mathrm{Sosm}={\left(\mathrm{Sosm}\left(\mathrm{peak}\right)-\mathrm{Sosm}\left(\mathrm{baseline}\right)\right)}^{\mathrm{Kt}}+\mathrm{Sosm}\left(\mathrm{baseline}\right) $$

S_osm_ = Salivary osmolality; S_osm(baseline)_ = Salivary osmolality at baseline (approximate; calculated as plateau phase of best fit model); S_osm(peak)_ = Salivary osmolality at peak (S_osm_ at end of Dehydration Protocol). Time was adjusted such that the time at S_osm(peak)_ was set to t = 0 in order to normalize fit parameters. K = rate constant, tau (τ) =1/K, and τ _1/2_ = half-time S_osm_ recovery. Performance loss was calculated as percent loss from baseline; performance recovery was calculated as a percentage of baseline peak torque where post-exercise peak torque was divided by baseline peak torque and expressed as a percentage (Peak Torque_post-ex_/Peak Torque_base_ X 100).

We used *p* values of < 0.05 to indicate statistical significance. Statistical calculations were calculated using commercially available software (GraphPad Prism version 5.0 for Mac OS X). Differences between averaged age, height, heart rate, BW, BMI, BSA, tympanic temperature, time to 3% body mass loss and sweat rate were determined using 2-way ANOVA with post-hoc Bonferroni analysis. All other comparisons were completed using a repeated measures 2-way ANOVA followed by a post-hoc Bonferroni analysis. No non-parametric tests were necessary, as all data were normally distributed.

## Results

### Age and body Morphometrics

At the time of the study, female participants (*n* = 8) were 22.1 ± 2.1 years of age, which was not different from male participants (*n* = 9) who were 23.6 ± 2.2 years of age. Female participants were significantly less in height when compared to male counterparts (167.6 ± 2.9 cm vs 181.1 ± 4.6 cm). Considering the significant difference in height, BW (65.2 ± 10.2 kg vs. 76.0 ± 8.6 kg) and BSA (1.74 ± 0.11 m^2^ vs 1.95 ± 0.14 m^2^) were significantly less in females compared to males. However, this difference was eliminated in the calculated BMI; females had a BMI of 23.3 ± 3.0 kg/m^2^ while males had a BMI of 23.2 ± 3.4 kg/m^2^.

### Heart rate and body temperature

Although baseline heart rate trended higher in females (85.8 ± 6.4 bpm) compared to males (80.4 ± 16.3 bpm), the trend did not reach significance. For each 15-min bout of exercise, we recorded peak heart rate and subsequently averaged these values to arrive at a single peak heart rate. We saw no significant impact of sex on peak heart rate, and no interaction between sex and exercise on peak heart rate.

Tympanic temperature as an indicator of core temperature was also recorded throughout the exercise protocol. Despite being subjected to exercise and moderate heat stress, both female (97.9 ± 0.5 °F vs. 99.2 ± 0.7 °F) and male (97.7 ± 0.8 °F vs. 99.3 ± 0.7 °F) participants maintained body temperature to a similar extent, displaying no significant increase in core temperature during exercise.

### Exercise and salivary osmolality

**Fig. 2:**
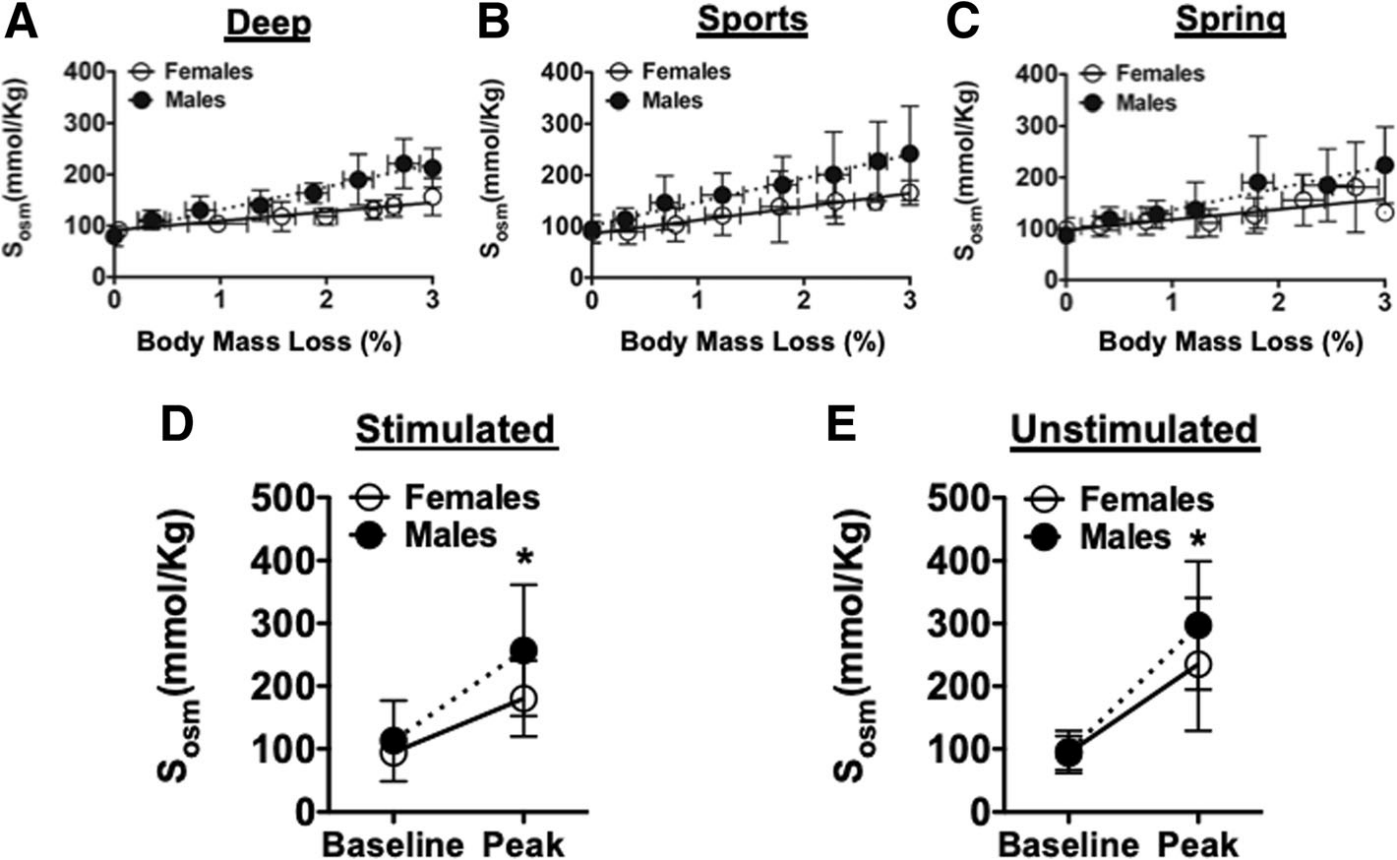
Salivary osmolality as a function of body mass loss. Salivary osmolality (S_osm_ mmol/Kg) was plotted as a function of change in body mass percentage in each of the three groups: Deep (**a**), Sports (**b**) or Spring (**c**) groups. Individual measures of salivary osmolality were averaged from the three trials. Salivary osmolality (S_osm_ mmol/Kg) was significantly elevated (*p < 0.0001; Females and Males) at Peak compared to Baseline collected as either Stimulated (**d**) or Unstimulated (**e**). No differences between Females and Males were detected, nor was there an interaction between sex and time point of data collection. Two-way ANOVA with post-hoc Bonferroni analysis. Data presented as mean ± S.D

For each saliva sample (unstimulated and stimulated), we determined salivary osmolality (S_osm_) and plotted S_osm_ against the percent of body mass lost. For display purposes, we represent the data as binned samples ± standard deviation (S.D.). Stimulated and unstimulated S_osm_ were significantly (positively) correlated with percent of body mass loss for both females and males. As illustrated in Fig. 2, S_osm_ progressively increased during exercise bouts paralleling lost body mass. The relationship of S_osm_ and percent body mass loss was not different between females and males.

No significant differences in baseline S_osm_ among study groups based on fluid designation were detected, validating that participants began each arm of the three trials at a similar hydration level. Baseline S_osm_ was not effected by sex in the stimulated (females 94.39 ± 14.90 vs males 113.00 ± 63.84), or the unstimulated (females 94.06 ± 26.62 vs males 95.51 ± 33.27) saliva samples, and there was no interaction between study group designation and sex. Peak S_osm_ (stimulated and unstimulated) was taken as the value for S_osm_ once participants reached at least 3% body mass loss. Similar to baseline, peak S_osm_ was not significantly impacted by either study group designation or sex in the stimulated (females 180.29 ± 60.37 vs males 256.96 ± 104.57), or the unstimulated (females 235.04 ± 105.99 vs males 297.14 ± 102.39) saliva samples. Moreover, there was no significant interaction between these factors on peak S_osm_. At the completion of 3% body mass loss, all participants achieved a significant (*p* < 0.0001) increase in averaged S_osm_ over baseline values. However, this elevation in S_osm_ was not affected by the sex of the participant (Fig. 2d, e).

### Time to body mass loss

**Fig. 3:**
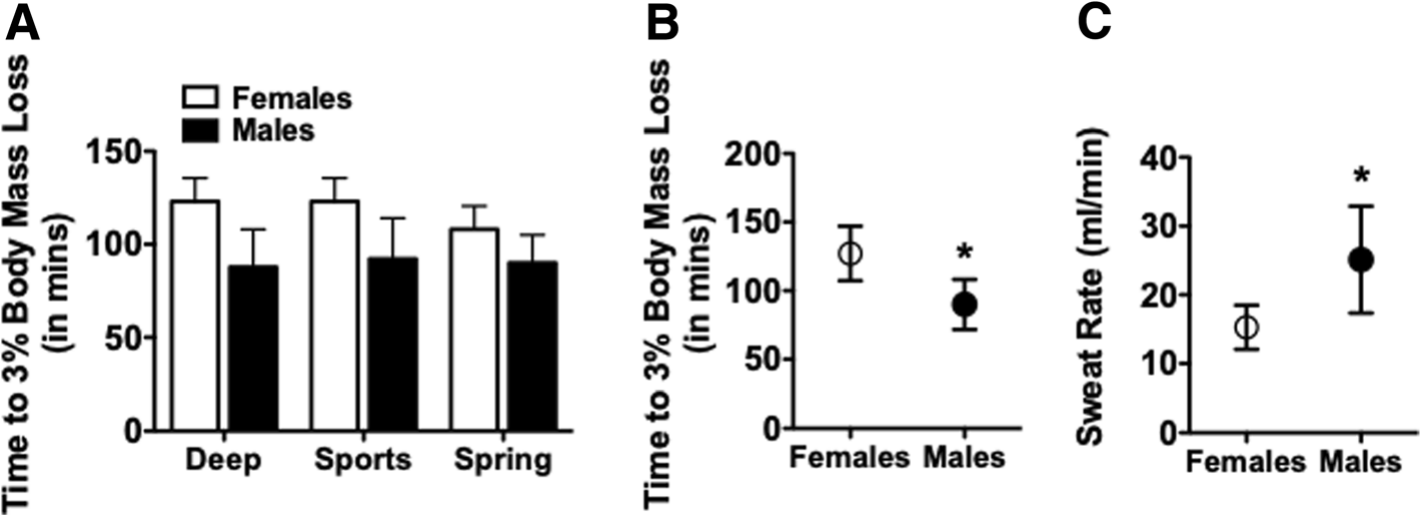
Time required to achieve target dehydration indicated by a 3% body mass loss. **a** Bar graph representation of the time required to reach a loss of body mass that was at least 3% of starting body mass in the Deep, Sports and Spring groups for Females and Males. A repeated measures two-way ANOVA indicated no significant differences in Time to 3% Body Mass Loss among the experimental groups but did find a significant effect of sex (*p* < 0.0125). **b** Averaged values across experimental groups for Time to 3% Body Mass Loss in Females and Males. (*p < 0.034; Females vs. Males). **c** Averaged values across experimental groups for Sweat Rate (ml/min; total volume lost in ml over total time to 3% body mass loss;) in Females and Males. (*p < 0.0083; Females vs. Males). Two-way ANOVA with post-hoc Bonferroni analysis. Data presented as mean ± S.D

We compared the expended time for participants to achieve a 3% body mass loss in each study arm (study group designation). Participants (female and male) did not differ in the time to achieve a 3% body mass loss between the 3 trials (Fig. 3a). We found a significant effect (*P* < 0.0125) of sex on time to achieve 3% body mass loss, with no interaction between study group designation and sex. Subsequent comparison of the mean values for each participant demonstrated that males took less time (90.0 ± 18.3 min; *P* < 0.0034) to reach the required body mass loss when compared to females (127.1 ± 20.0 min) (Fig. 3b). Assuming that body mass loss during the exercise period was due to water loss, we calculated sweat rate using the absolute mass of lost body mass and the total time to reach 3% body mass loss (Fig. 3c). Based on the averaged values of sweat rate for each participant, females (15.3 ± 3.2 ml/min; *p* < 0.0083) demonstrated lower sweat rates compared to males (25.2 ± 7.8 ml/min), consistent with the greater time to achieve 3% body mass loss in the female group.

### Impact of fluid on S_osm_ recovery (rehydration)

**Fig. 4:**
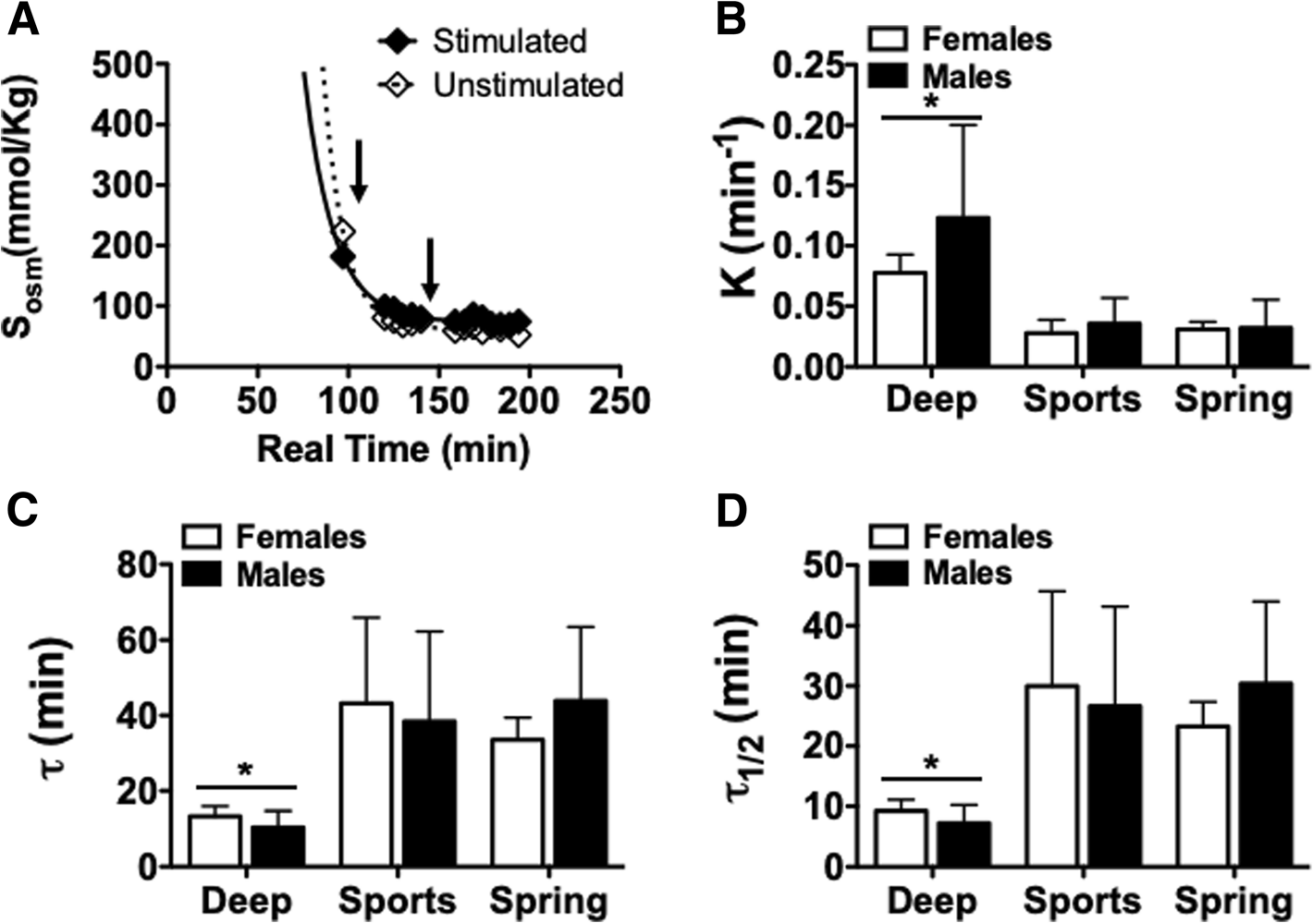
Rate of salivary osmolality recovery during fluid hydration following dehydrating exercise protocol. Salivary osmolality was fit with a single exponential decay (one-phase decay) starting with peak salivary osmolality against real time. **a** representative one-phase decay fit to salivary osmolality recovery during fluid hydration. Fluid was ingested in two phases indicated by the arrows. **b**-**d** Bar graph representation of one-phase decay fit parameters (K, tau (τ), and τ _1/2_) in Females and Males from the Deep, Sports and Spring groups. A repeated-measures two-way ANOVA determined a significant impact of fluid on rate parameters of hydration that was not impacted by sex. Post-hoc Bonferroni analysis indicated a significant difference in the Deep group compared to both Sports and Spring groups (*p < 0.0001 compared to Sports and Spring groups). One-phase decay equation: S_osm_ = (S_osm(peak)_- S_osm(baseline)_)^Kt^ + S_osm(baseline)_; K = rate constant, tau (τ) =1/K, and τ _1/2_ = half-time S_osm_ recovery. Data presented as mean ± S.D

Peak S_osm_ steadily declined and returned to baseline S_osm_ values before completion of the saliva collection time during the rehydration phase. We determined the rate constant (K), time constant (τ), and the time constant half-life (τ _1/2_) of this decay for each participant in each trial (Fig. 4a). We found a significant effect of rehydrating fluid (*p* < 0.0001) that was not impacted by sex. Post-hoc analysis revealed a significantly higher K, and a significantly lower τ, and τ _1/2_ in the **Deep** than in both the **Sports** and **Spring** groups in both females and males (Figs. 4b-d, values in Table 2). The same trend of significance was seen whether S_osm_ was taken from the stimulated or the unstimulated samples.

**Table 2:** The return of S_osm_ to baseline levels during rehydration

	Recovery of Salivary Osmolality (S_osm_)		
		Females (n = 8)	Males (n = 9)
Stimulated S_osm_			
Deep			
K (min^−1^)		0.077 ± 0.015	0.123 ± 0.077
τ (min)		13.3 ± 2.6	10.5 ± 4.3
τ _1/2_ (min)		9.2 ± 1.8	7.2 ± 3.0
Sports			
K (min^− 1^)		0.028 ± 0.011	0.035 ± 0.021
τ (min)		43.2 ± 22.7	38.5 ± 23.8
τ _1/2_ (min)		30.0 ± 15.8	26.6 ± 16.5
Spring			
K (min^− 1^)		0.031 ± 0.006	0.032 ± 0.023
τ (min)		33.6 ± 5.9	43.9 ± 19.6
τ _1/2_ (min)		23.3 ± 4.1	30.4 ± 13.6
Unstimulated S_osm_			
Deep			
K (min^− 1^)		0.085 ± 0.017	0.106 ± 0.0.38
τ (min)		12.3 ± 2.7	10.3 ± 2.7
τ _1/2_ (min)		8.5 ± 1.8	7.2 ± 1.8
Sports			
K (min^−1^)		0.028 ± 0.013	0.030 ± 0.014
τ (min)		42.0 ± 16.6	32.9 ± 9.6
τ _1/2_ (min)		29.1 ± 11.5	22.8 ± 6.6
Spring			
K (min^−1^)		0.028 ± 0.011	0.032 ± 0.023
τ (min)		42.1 ± 13.3	40.0 ± 16.1
τ _1/2_ (min)		29.1 ± 19.1	27.7 ± 11.2

The relationship between S_osm_ and time during the rehydration phase was best fit by a mono-exponential (one-phase decay) model. One-phase decay equation: Sosm = (Sosm(peak)- Sosm(baseline))Kt + Sosm(baseline); K = rate constant, tau (τ) =1/K, and τ _1/2_ = half-time S_osm_ recovery. Data presented as mean ± S.D

### Dehydration, rehydration, and exercise performance

**Fig. 5:**
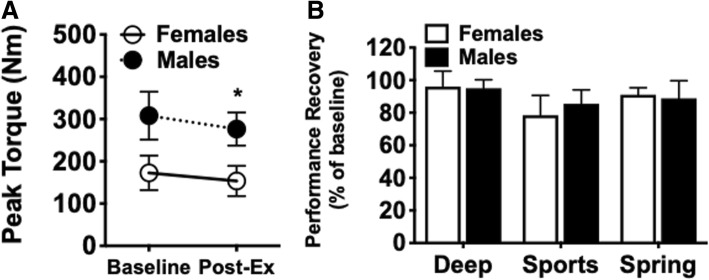
Impact of dehydration and hydration on lower body muscle performance. Peak torque extension using Biodex™ was determined prior (Baseline) to and immediately following exercise (Post-Ex) and rehydrating protocol. **a** Averaged values across experimental groups for peak torque extension (Nm) at Baseline and Post-Ex in Females and Males. The dehydrating exercise protocol significantly impacted peak torque (*p* = 0.0048), which was dependent on sex (*p* = 0.0005) and the exercise protocol (*p* = 0.0002) by two-way ANOVA. Post-hoc Bonferroni analysis indicated a significant difference in Baseline and Post-Ex for Males only (*p* = 0.0117). the Deep group compared to both Sports and Spring groups (*p = 0.01; Females vs. Males). **b** Bar graph representation of the recovery in peak torque extension at the completion of the hydration protocol expressed as percentage of baseline peak torque (Peak Torque_end_/Peak Torque_base_ X 100). There is a main effect of rehydrating fluid (*p* = 0.0157) by two-way ANOVA. Data presented as mean ± S.D

Each participant (female and male) achieved a similar peak torque extension at baseline and immediately following 3% body mass loss across each study arm and is summarized in Fig. 5a. The dehydrating exercise protocol significantly impacted peak torque (*p* = 0.0048), which was dependent on sex (*p* = 0.0005). Overall, males generated greater peak torque extension at baseline when compared to females (308.3 ± 56.7 Nm vs 172.8 ± 40.8 Nm, respectively) and immediately following loss of 3% body mass (276.3 ± 39.5 Nm vs 153.5 ± 35.9 Nm, respectively). When comparing baseline and post-exercise peak torque, there was significant main effect of the exercise protocol on peak torque (*p* = 0.0002). However, the loss of peak torque reached significance only in males (9.6 +/− 7.4%; *p* = 0.0117) and not in females (10.6 +/− 8.1%; *p* = 0.1998). Although participants in each study group failed to fully recover peak torque extension following rehydration, there was a significant effect of the rehydrating fluid (*p* = 0.0157) on the percent peak torque recovery (Fig. 5b).

## Discussion

The goal of the study was to evaluate parameters of dehydration and associated performance deficits due to dehydrating exercise, and then to determine if hydration and muscle performance recovery was dependent on fluid type. Secondarily, we observed potential sex differences in these parameters, although the study was not explicitly powered for such comparisons.

### Heart rate and body temperature

Our observations on increases in heart rate are consistent with most [21–24], but not all [25] studies in the literature reporting statistically similar increases in heart rate for females and males during strenuous exercise. It has been suggested that males and females may differ in heart rate response to exercise, due in part to differences in exercise capacity, with men being able to reach higher exercise intensities, and therefore generate larger changes in heart rate during exercise [25]. It has also been suggested that a bias may exist in research personnel against pushing females as hard as males during exercise [25], and that males may put in a higher degree of effort during exercise than females [22], both of which could show confounded sex differences in peak heart rate. Indeed, we informally observed that males tended to exercise at a higher workload than females during our exercise study. However, our study, as well as another [22] showed similar max heart rates in females as males, despite the appearance of a difference in effort, indicating that males and females demonstrated similar exertion.

Sex differences may exist in thermoregulatory factors following heat stress and exercise [26], and we kept track of participants’ tympanic temperature, which is representative of body temperature [27], throughout the exercise protocol. We observed a slight but statistically insignificant increase in tympanic temperature throughout the duration of the exercise protocol in men and women, with no differences between the sexes. This lack of difference between sexes was not surprising, because although males and females differ in some specific aspects of thermoregulation (sweat rate and evaporative cooling efficiency) during exercise in the heat, it is thought that females and males are able to maintain body temperature with similar efficiency [26]. However, we did not expect to see an overall lack of significant increase in body temperature after exercise, since much of the literature supports the idea that exercise, heat, and dehydration impair thermoregulation [3, 11, 26]. One potential explanation for our data may be variation in the duration to achieve 3% body mass loss [3] or the fitness level of the study participants [3, 28]. More likely, acclimation to exercising in hot conditions may be the reason for this observation. Heat acclimation may provide the athlete with the benefit of expanded erythrocyte volume, and plasma volume, both of which have the potential to improve thermoregulatory ability in athletes [29]. We did not account for heat acclimation in this study, but it is reasonable to infer that some or all of the study participants had some level of heat acclimation living in Arizona, a region with a hot, dry climate throughout most of the year.

### Salivary osmolality and time to body mass loss

Average baseline S_osm_ was not different between males and females. As expected, the total time to achieve a 3% reduction in body mass was similar among the three trials completed by each participant, regardless of sex, indicating consistency in exercise performance. Furthermore, we confirmed a significant positive correlation between percent body mass loss through sweat (dehydration) and S_osm_ for both males and females, as expected during intense exercise in the heat. These were important observations, because they indicate that participants started at the same hydration level and executed a similar amount of exercise during each trial. Although power output was not measured, we observed that men may have had higher average power output and tended to use greater resistance throughout the workout, consistent with findings showing higher aerobic workload capacity in men compared to women [30]. A higher power output in males could be one reason for the observed shorter time-to-dehydration than females. This difference in time-to-dehydration could also be attributed to a faster general sweat rate in males than in females, mainly due to greater body surface area and lower surface area-to-mass ratio, and greater metabolic heat production in males than in females [30, 31]. Although females generally have a greater number and density of eccrine sweat glands than men [30], the per-gland sweat secretion rate is a larger contributing factor to overall sweat rate than the number or density of sweat glands [31]. Sweat secretion rate per gland varies inter- and intra-individually, but it is possible that this factor may be partially responsible for this observed sweat rate difference.

### Dehydration, rehydration, and exercise performance

Baseline and post-exercise values indicated that males generated greater peak torque than females, as expected, based on a higher average muscle mass in males than in females. In our study, fluid loss due to exercise resulted in a significant muscle performance deficit that was not impacted by sex. Although current literature is fairly inconclusive, results from many studies do suggest that dehydration negatively impacts muscular strength, power, and endurance [32]. However, there is relatively little research comparing potential dehydration-induced decline in muscle strength between men and women, and results of such studies vary. [32, 33]. The results of the current study do not necessarily support the notion that dehydration negatively impacts muscle strength, as the effects of dehydration were not isolated from the effects of exercise and muscle fatigue in this study.

Interesting findings from previous work suggest that consumption of deep-ocean mineral water following a dehydrating exercise protocol improves aerobic performance and muscle strength [13, 14]. Although these studies did not measure “hydration status”, our initial study demonstrated a significantly more rapid return to pre-exercise (baseline) hydration levels in addition to improved recovery of lower body muscle performance following rehydration (post-hydration) with deep-ocean mineral water [15]. In this more comprehensive study, we found that male and female participants demonstrated elevated rates of hydration recovery, and that peak torque of a leg extension may also be improved when fluid was replenished with deep-ocean mineral water compared to other fluids. Therefore, improved acute hydration may be one factor by which deep-ocean mineral water improves exercise performance, as has been shown.

Although we did not study the precise mechanism underlying enhanced fluid recovery with deep-ocean mineral water, it is likely that the unique mineral composition of deep-ocean mineral water contributes to this characteristic (See Table 1 for a nutrient comparison of fluids). A 2013 study by Hou et al. [13] speculates that elevated levels of magnesium (Mg) in deep-ocean mineral water may be a major contributor to its performance-enhancing effects, citing evidence of a correlation between Mg status and muscle strength. However, Kona Deep® contains far less Mg than the deep-ocean mineral water used in the Hou study, and therefore, we cannot necessarily predict that the modest difference in Mg between the three fluids in our study was a major contributor to the observed effects on muscle performance. Additionally, we have no evidence to support a connection between Mg and hydration recovery.

Another possible mineral contributor is boron. Both Kona Deep® and the water used in the Hou study contain significant amounts of this trace mineral. Hou reports that boron attenuates the rise in plasma lactate, potentially delaying fatigue, and prevents Mg loss. As with Mg, however, we have no evidence to support a connection between boron and hydration recovery. Interestingly, composition of the intake fluid impacts intestinal water flux more so than osmolality [34]. Carbohydrate-electrolyte sports drinks, such as Gatorade®, are proposed to increase intestinal water absorption due to the presence of glucose, which assists sodium transport into the intestinal cells via the sodium-glucose cotransporter, thereby influencing water flux by promoting an osmotic gradient [35, 36]. However, we observed no greater acute hydration rate with Gatorade® compared to the other fluids. This may be due to the influence of gastric emptying rates, as fluids containing carbohydrates may decrease gastric emptying rate compared to non-carbohydrate-containing fluids [36, 37]. Notably, slower gastric emptying rates may also decrease intestinal absorption rates [35], thereby slowing overall fluid uptake and assimilation into the body fluid compartments.

### Limitations

Several limitations of the study have been mentioned throughout the paper. We relied on the use of salivary osmolality as the sole marker of hydration throughout the study. Previous work shows that salivary osmolality is highly valuable for serial measures of hydration during intense physical activity in the heat [18]. More importantly, we needed multiple data points to best model instantaneous changes in osmolality throughout the dehydration and rehydration periods. Due to the continuous nature of the exercise protocol, serial urine collections were not practical for this study. Some limitations do exist for the use of S_osm_ as a marker of hydration, including an initial sharp drop in osmolality caused by oral rinse and variability between participants [3, 15, 17–19]. To account for these limitations, saliva samples were collected starting at 10 min after participants ingested the rehydrating fluid, which allowed S_osm_ to return to pre-rinse levels. Furthermore, baseline, peak or the rate of increase in S_osm_ across the 3 trials was similar for each participant, indicating S_osm_ was an appropriate method for comparing rehydration fluids *within* each participant.

Participants were separated by sex based on secondary analysis of study parameters. Because the study was not powered for sex differences, analysis of peak torque would require further studies specifically powered for sex as a primary outcome. Similarly, dietary restrictions were suggested and not strictly enforced and cannot be ruled out as a potential contributor to any sex differences.

Finally, the American College of Sports Medicine (ACSM) recommends 1.25–1.5 L of replacement fluid for every 1 kg lost during exercise, as fluid loss continues post-exercise as a result of continued sweating and urinary losses [12]. In our study, participants replaced fluid lost in a 1:1 ratio. During development of the protocol in pilot studies, participants were not able to ingest fluid amounts suggested by the ACSM recommendations. In addition, participants did not urinate during rehydration, and all subjects completed the final saliva collection and muscle strength measurement at their full baseline body mass. Future studies will be designed to address these limitations as well as the underlying mechanisms by which deep-ocean mineral water elicited enhanced hydration effects, including the contribution of specific nutrients specific to deep-ocean mineral water.

## Conclusions

Kona Deep® deep-ocean mineral water improved acute rehydration rate after a dehydrating exercise in both males and females, compared to spring water and Gatorade®. However, it remains unclear whether the hydration-enhancing effect of deep-ocean mineral water impacts performance recovery as demonstrated previously [13–15]. Future studies will be targeted at uncovering the mechanisms behind the hydration-enhancing properties of deep-ocean mineral water, further characterizing sex differences in these relationships, and correlating additional measures of hydration, such as serum osmolality, with that of S_osm_.

## Abbreviations

BMI: Body Mass Index
bpm: Beats Per Minute
BSA: Body Surface Area
BW: Body Weight
CFTR: Cystic Fibrosis Transmembrane Protein
Deep: Kona Deep® Deep-Ocean Mineral Water
ECF: Extracellular Fluid
K: Rate Constant
S_osm_: Salivary Osmolality
Sports: Gatorade® Sports Drink
Spring: Arrowhead Mountain Spring Water
UAHHSQ: University of Arizona Health History Screening Questionnaire
τ: Time Constant
τ_1/2_: Time Constant Half-Life
